# Supplementary material for: NPAS4 refines spatial and temporal firing in CA1 pyramidal neurons
Source: bioRxiv. 2026 Mar 22:2026.03.22.713468. Preprint. [Version 1] doi: 10.64898/2026.03.22.713468 (PMC13015365; doi:10.64898/2026.03.22.713468)
Supplement: 1 [file NIHPP2026.03.22.713468V1-supplement-1.pdf]

bioRxiv preprint doi: https://doi.org/10.64898/2026.03.22.713468; this version posted March 22, 2026. The copyright holder for this preprint (which was not certified by peer review) is the author/funder, who has granted bioRxiv a license to display the preprint in perpetuity. It is made available under aCC-BY 4.0 International license.

1232 # Supplemental Figures

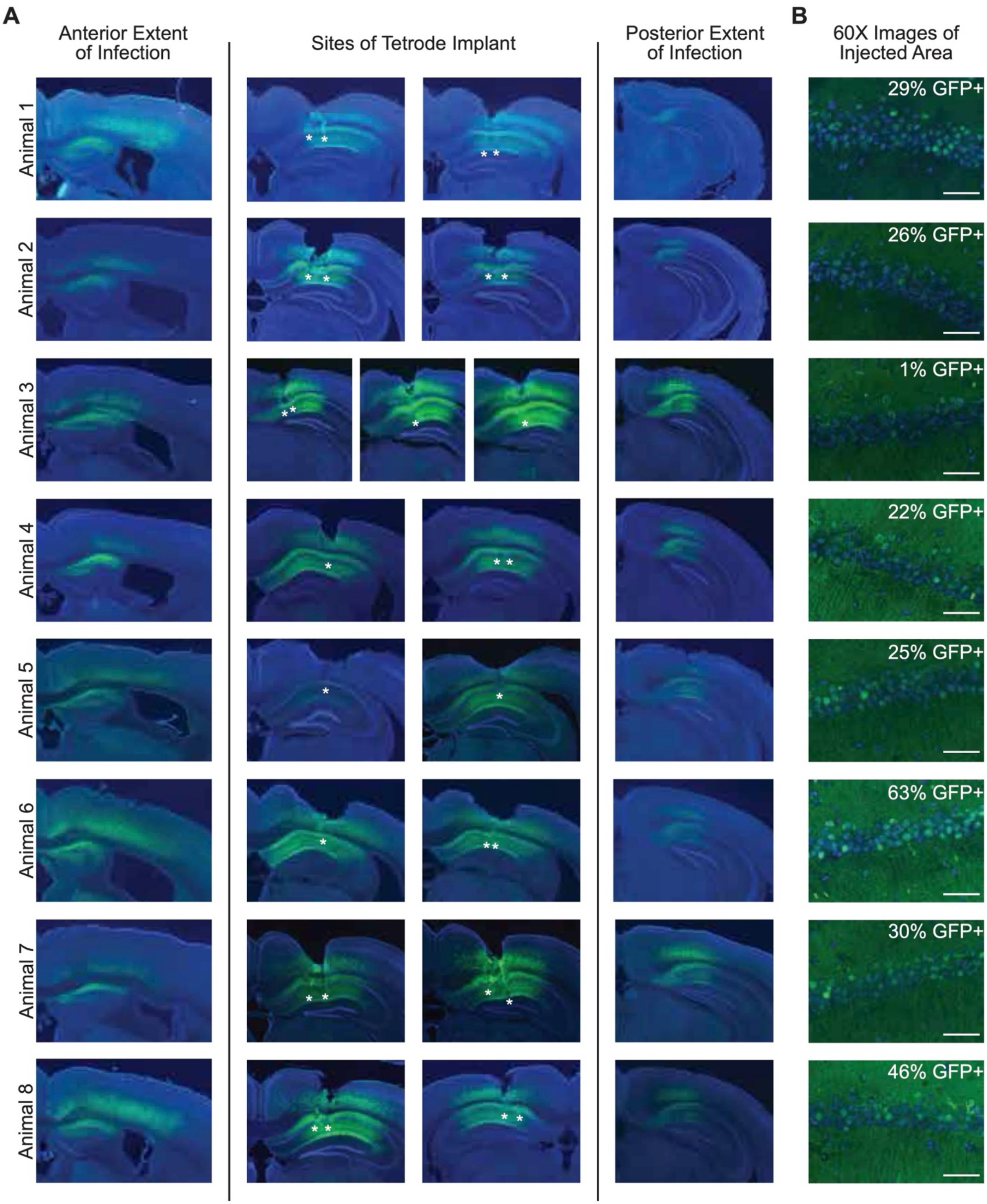

bioRxiv preprint doi: https://doi.org/10.64898/2026.03.22.713468; this version posted March 22, 2026. The copyright holder for this preprint (which was not certified by peer review) is the author/funder, who has granted bioRxiv a license to display the preprint in perpetuity. It is made available under aCC-BY 4.0 International license.

Supplemental Figure 1. Representative histology for all sparse-infection animals

(A) Left: Stitched 10X images showing the anterior extent of the infection. Middle: Site of optetrode implant in CA1, asterisks represent the end of the tetrode tracts. Not all tetrode tracts were able to be identified. Right: Stitched 10X images showing the posterior extent of the infection.

(B) Representative 60X images near the site of implant showing sparse infection. Scale bar = 50 μm.

bioRxiv preprint doi: https://doi.org/10.64898/2026.03.22.713468; this version posted March 22, 2026. The copyright holder for this preprint (which was not certified by peer review) is the author/funder, who has granted bioRxiv a license to display the preprint in perpetuity. It is made available under aCC-BY 4.0 International license.

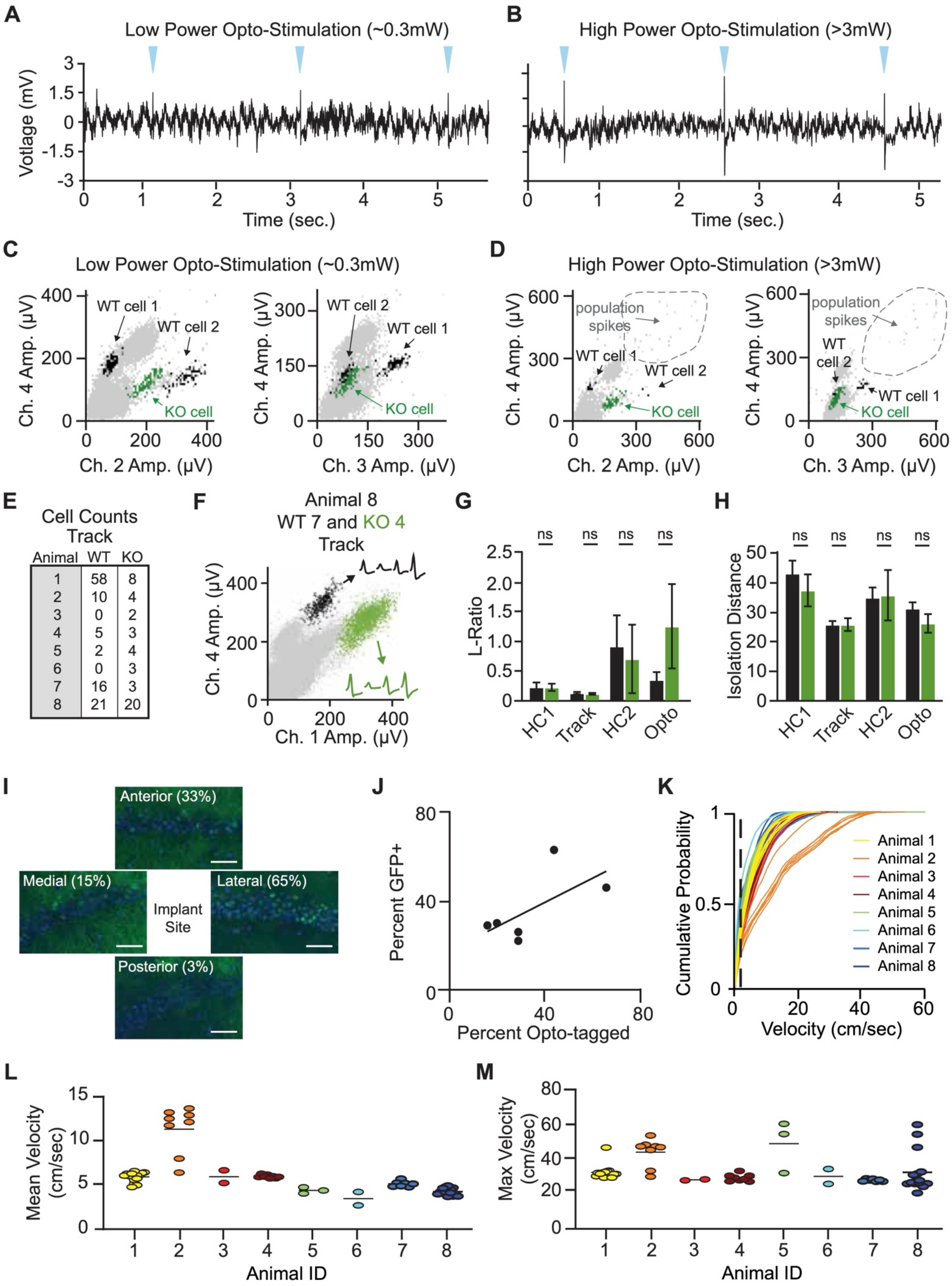

bioRxiv preprint doi: https://doi.org/10.64898/2026.03.22.713468; this version posted March 22, 2026. The copyright holder for this preprint (which was not certified by peer review) is the author/funder, who has granted bioRxiv a license to display the preprint in perpetuity. It is made available under aCC-BY 4.0 International license.

Supplemental Figure 2. Optical identification and functional characterization of NPAS4 KO neurons in vivo

(A) Example of unfiltered LFP recording during light stimulation. Blue triangles = light delivery.
(B) Example of unfiltered LFP recording during high-power light stimulation.
(C) Example of cluster cutting for two WT neurons and a KO neuron during low-level light stimulation. Black dots: spikes from two WT neurons; green dots: spikes from one KO neuron.
(D) Same neurons and cluster views as in (C) but for high-power stimulation. Dotted circle: putative population spikes.
(E) WT and KO cell counts for each animal during track recordings.
(F) Example of cluster cutting for a track recording showing a WT cell (black) and KO cell (green). Insets are average spike waveforms for each of the four tetrode channels for each neuron.
(G) L-ratio for WT and KO cells for pre-track home cage (HC1), track, post-track home cage (HC2), and during optostimulation (HC1 WT N=70, KO N=34; track WT N=112, KO N=36; HC2 WT N=78, KO N=15; opto WT N=108, KO N=33; Kolmogorov–Smirnov test).
(H) Isolation distance for WT and KO cells for HC1, track, HC2, and opto (HC1 WT N=71, KO N=36; track WT N=111, KO N=37; HC2 WT N=78, KO N=16; opto WT N=108 KO N=34; Kolmogorov–Smirnov test).
(I) Example of how the percent infection is obtained from histology. 60X images taken anterior, posterior, medial, and lateral of the implant site. The percent infection per animal shown in (J) is an average of all four sites. Scale bar = 50 μm.
(J) The percent of infected cells identified in histology plotted against the percent of optotagged cells identified *in vivo*. Line of best fit is shown.
(K) Distribution of velocity for each session the sparse KO animals ran.
(L) Mean velocity for all sparse KO animals.
(M) Max velocity for all sparse KO animals.
ns = not significant.

bioRxiv preprint doi: https://doi.org/10.64898/2026.03.22.713468; this version posted March 22, 2026. The copyright holder for this preprint (which was not certified by peer review) is the author/funder, who has granted bioRxiv a license to display the preprint in perpetuity. It is made available under aCC-BY 4.0 International license.

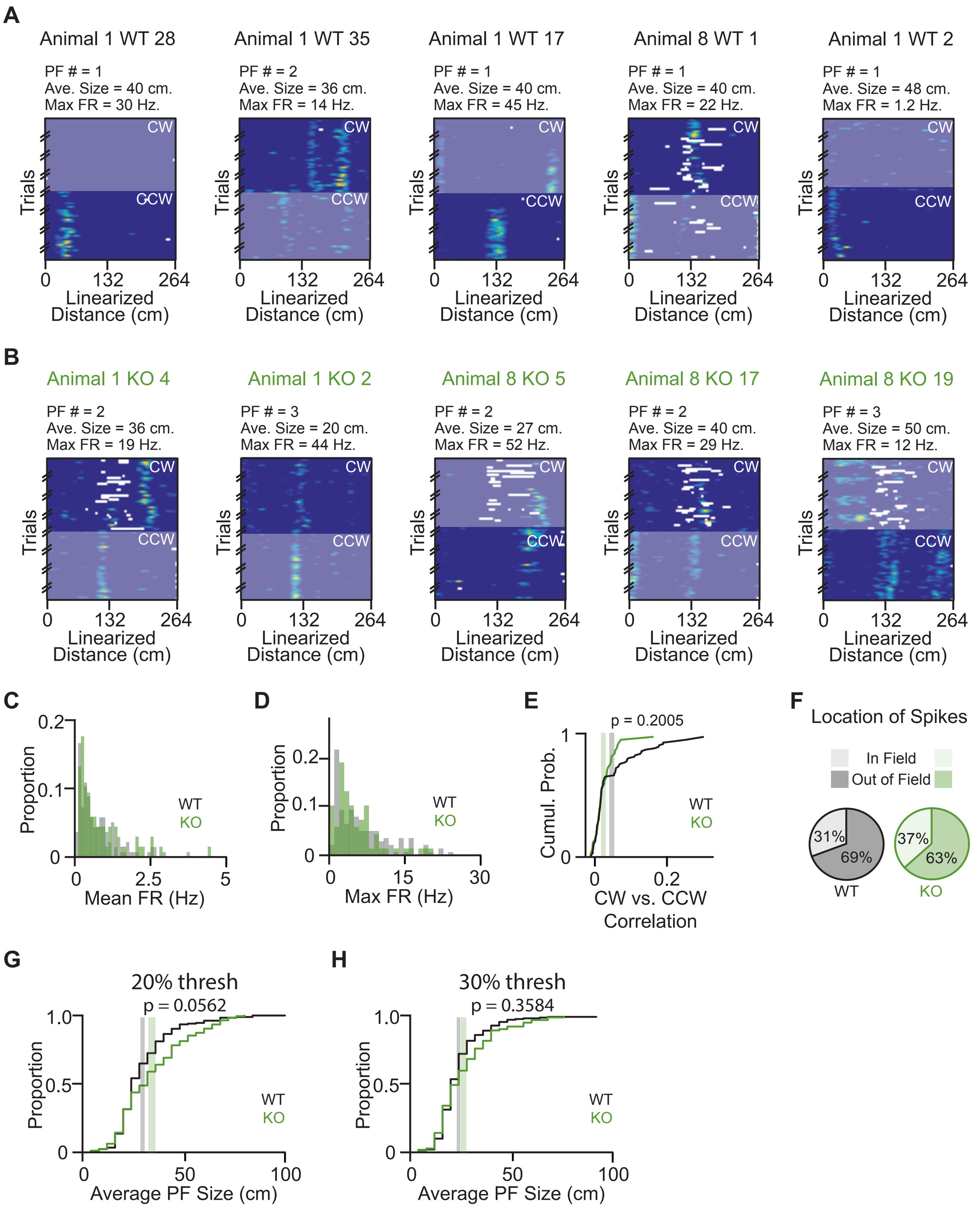

bioRxiv preprint doi: https://doi.org/10.64898/2026.03.22.713468; this version posted March 22, 2026. The copyright holder for this preprint (which was not certified by peer review) is the author/funder, who has granted bioRxiv a license to display the preprint in perpetuity. It is made available under aCC-BY 4.0 International license.

Supplemental Figure 3. Additional information on spatial firing rate properties for NPAS4 WT and KO neurons

(A) Additional examples of rate maps from five WT cells. For each cell, both clockwise (CW) and counterclockwise (CCW) directions are shown; the direction not used for analysis is greyed out. The summary data shown above each rate map (Place Field Number [PF #], Average Size [Ave. Size], and Maximum Firing Rate [Max. FR]) corresponds to the analyzed direction only.

(B) As in (A) but for KO cells.

(C) Histogram of mean firing rate for all neurons (both low firing and high firing; WT: N = 224; KO: 94).

(D) As in (C) but for the maximum firing rate.

(E) Pearson's Correlation Coefficient (PCC) between the trial-averaged CW rate map for each neuron and the trial-averaged CCW rate map (WT: N = 138; KO: N = 68; Kolmogorov–Smirnov test).

(F) The percentage of all spikes for all neurons that occurred in-field and out-of-field for WT and KO neurons (WT: N = 140; KO: N = 68; p = 0.21; chi-square goodness-of-fit test).

(G) Average place field size as calculated in the main figure with the exception that the minimum threshold used was 20% of max firing instead of 10% of max firing (WT: N = 138; KO: N = 68; Wilcoxon rank sum test).

(H) As in (G) but with a minimum threshold of 30% (WT: N = 138; KO: N = 68; Wilcoxon rank sum test).

bioRxiv preprint doi: https://doi.org/10.64898/2026.03.22.713468; this version posted March 22, 2026. The copyright holder for this preprint (which was not certified by peer review) is the author/funder, who has granted bioRxiv a license to display the preprint in perpetuity. It is made available under aCC-BY 4.0 International license.

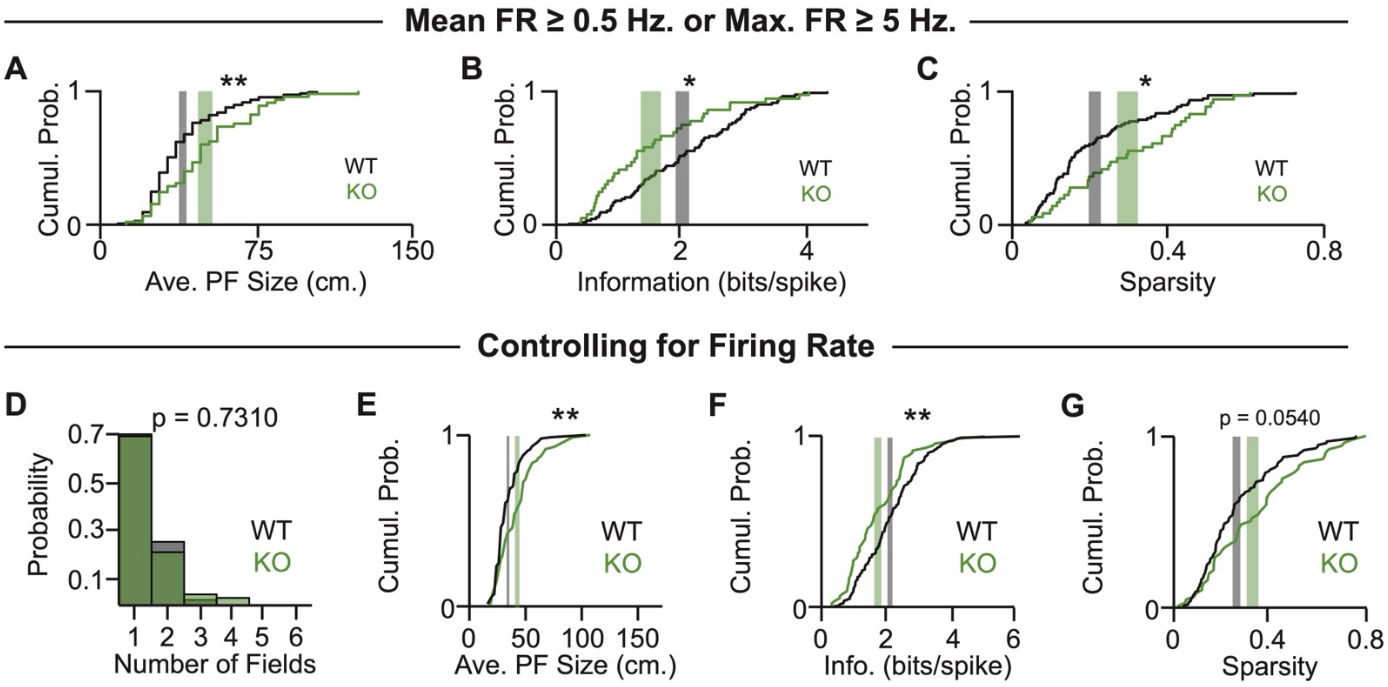

bioRxiv preprint doi: https://doi.org/10.64898/2026.03.22.713468; this version posted March 22, 2026. The copyright holder for this preprint (which was not certified by peer review) is the author/funder, who has granted bioRxiv a license to display the preprint in perpetuity. It is made available under aCC-BY 4.0 International license.

Supplemental Figure 4. Spatial deficits persist across firing rate thresholds, matched firing rates, and independent replication.

(A) Cumulative probability distribution of average place field size when only including neurons with mean firing rates > 0.5 Hz or max firing rates > 5 Hz. Gray shaded region: ± SEM for WT, centered at the mean; green shaded region: ± SEM for KO, centered at the mean (WT: N = 81; KO: N = 36; Kolmogorov–Smirnov test).

(B) As in (A) but for spatial information.

(C) As in (A) but for sparsity.

(D) Histogram of the number of place fields per neuron when the number of spikes is the same across all neurons (WT: N = 138; KO: N = 68; Mann–Whitney test).

(E) Cumulative probability distribution of average place field size when the number of spikes is the same across all neurons. Gray shaded region: ± SEM for WT, centered at the mean; green shaded region: ± SEM for KO, centered at the mean (WT: N = 81; KO: N = 36; Kolmogorov–Smirnov test).

(F) As in (E) but for spatial information.

(G) As in (E) but for sparsity.

*p < 0.05; **p < 0.01.

bioRxiv preprint doi: https://doi.org/10.64898/2026.03.22.713468; this version posted March 22, 2026. The copyright holder for this preprint (which was not certified by peer review) is the author/funder, who has granted bioRxiv a license to display the preprint in perpetuity. It is made available under aCC-BY 4.0 International license.

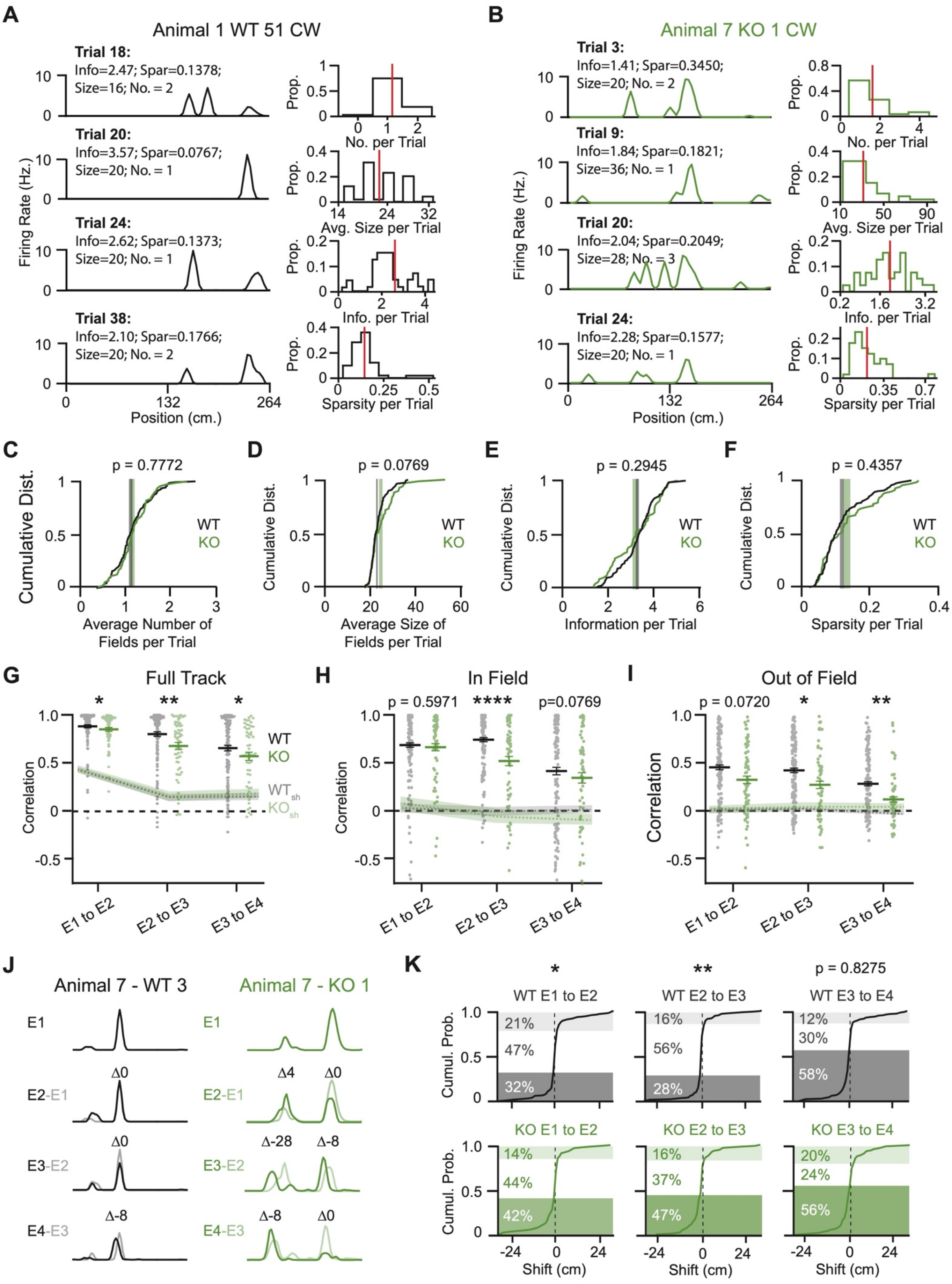

bioRxiv preprint doi: https://doi.org/10.64898/2026.03.22.713468; this version posted March 22, 2026. The copyright holder for this preprint (which was not certified by peer review) is the author/funder, who has granted bioRxiv a license to display the preprint in perpetuity. It is made available under aCC-BY 4.0 International license.

Supplemental Figure 5. NPAS4 stability deficits are the result of spurious out-of-field firing and shifts in the place field towards field entrance.

(A) Left: Four representative trials for a WT example cell. The number of fields, size, information, and sparsity are calculated independently for each trial. Right: For the same example cell, histograms for (top to bottom) the number of place fields, the average size of the place fields, the information, and the sparsity calculated for each trial independently and shown for all trials. Red line depicts the average.

(B) As in (A) but for an example KO cell.

(C) Median number of place fields per neuron. The number of fields was computed independently for each trial, then the median value was calculated for each neuron (WT N=140, KO N=68; Mann-Whitney test).

(D) As in (C) but for the average place field size (WT N=140, KO N=68; Kolmogorov–Smirnov test).

(E) As in (C) but for the spatial information (WT N=140, KO N=68; Kolmogorov–Smirnov test).

(F) As in (C) but for the sparsity (WT N=140, KO N=68; Kolmogorov–Smirnov test).

(G) Correlation between sequential sets of epochs (E1 to E2, E2 to E3, and E3 to E4) for all bins across the track. Gray dots: WT neurons; green dots: KO neurons; dotted gray: shuffled WT; dotted green: shuffled KO (WT N=140, KO N=68; Kolmogorov–Smirnov test).

(H) As in (G) but for only the in-field bins for each neuron (WT N=140, KO N=68; Kolmogorov–Smirnov test).

(I) As in (G) but for only the out-of-field bins for each neuron (WT N=140, KO N=68; Kolmogorov–Smirnov test).

(J) Example trial-averaged rate maps for each epoch from one WT (left) and one KO (right) neuron to show shift calculation. Shift (denoted as Δ) is negative when the field shifts towards field entrance and positive when it shifts towards field exit.

(K) Shift values for WT (top) and KO (bottom) across sequential sets of epochs (E2-E1, E3-E2, E4-E3). Dark shaded regions: fields with shift less than -1; unshaded regions: fields with shift between -1 and 1; light shaded regions: fields with shift greater than 1. Significance indicates comparisons between WT and KO (WT: N = 176 fields from 138 neurons; KO: N = 91 fields from 68 neurons).

*$p < 0.05$; **$p < 0.01$; ****$p < 0.0001$.

bioRxiv preprint doi: https://doi.org/10.64898/2026.03.22.713468; this version posted March 22, 2026. The copyright holder for this preprint (which was not certified by peer review) is the author/funder, who has granted bioRxiv a license to display the preprint in perpetuity. It is made available under aCC-BY 4.0 International license.

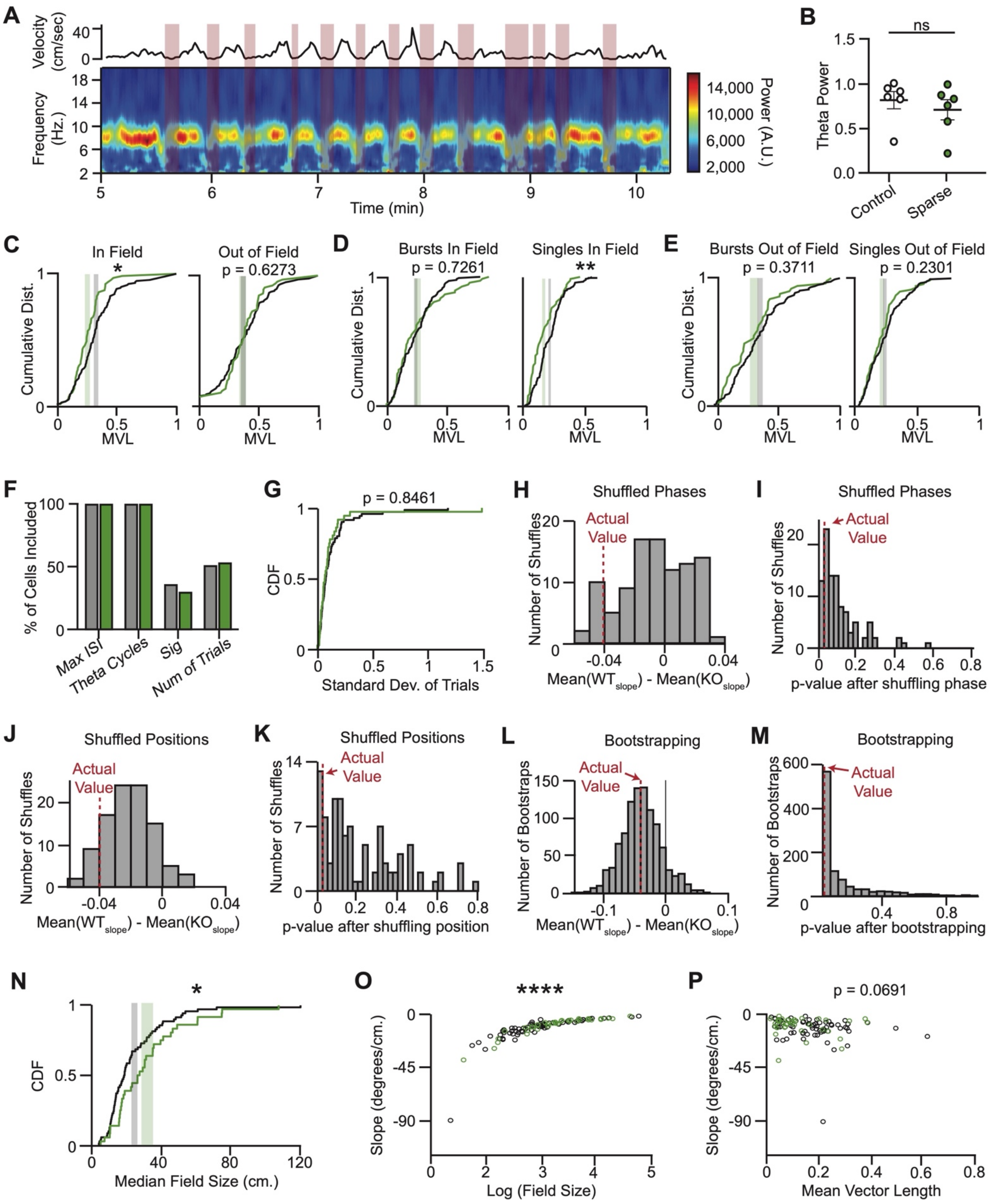

bioRxiv preprint doi: https://doi.org/10.64898/2026.03.22.713468; this version posted March 22, 2026. The copyright holder for this preprint (which was not certified by peer review) is the author/funder, who has granted bioRxiv a license to display the preprint in perpetuity. It is made available under aCC-BY 4.0 International license.

Supplemental Figure 6. Impaired theta modulation in NPAS4 KO neurons is carried by single spikes but not bursts and the accompanying phase precession phenotype is related to differences in the size of the place fields.

(A) Velocity (top) and spectrogram (bottom) for a representative session. Shaded red bars are periods of time when the velocity is below 2 cm/sec.

(B) Theta power after accounting for the aperiodic offset (data are mean ± SEM; control N=6 animals, sparse N=8; Kolmogorov–Smirnov test).

(C) Cumulative probability distribution of mean vector lengths for the in-field spikes (left) or out-of-field (right) spikes. Gray shaded region: ± SEM for WT, centered at the mean; green shaded region: ± SEM for KO, centered at the mean (WT: N = 138; KO: N = 68; Kolmogorov–Smirnov test).

(D) As in (F) but for spikes in a burst that occurred in-field (left) or singles (spikes not in a burst) that occurred in-field (WT: N = 138; KO: N = 68; Kolmogorov–Smirnov test).

(E) As in (F) but for spikes in a burst that occurred out-of-field (left) or singles (spikes not in a burst) that occurred out-of-field (WT: N = 138; KO: N = 68; Kolmogorov–Smirnov test).

(F) Percentage of neurons retained after sequential thresholding steps applied during phase precession slope estimation. Criteria included: 1. Max ISI - spikes within each trial must have interspike intervals ≤ 1 s; 2. Theta Cycles - trials must span ≥ 3 theta cycles; 3. Sig - trial-level circular-linear regression must yield a p-value < 0.05; 4. Number of Trials - neurons must have ≥ 3 trials meeting the above criteria to be included in downstream analyses (WT: N = 70; KO: N = 36).

(G) Cumulative probability distribution of the standard deviation of phase precession slopes across trials for WT and KO neurons. Gray shaded region: ± SEM for WT, centered at the mean; green shaded region: ± SEM for KO, centered at the mean (WT: N = 70; KO: N = 36; Kolmogorov–Smirnov test).

(H-K) Shuffle analyses to assess whether the group difference in phase precession slopes reflects structured relationships between spike phase and position.

(H) Histogram of mean slope differences (WT minus KO) across 100 iterations of theta phase shuffling, where theta phases were randomly permuted within each trial to disrupt spike–theta alignment. Red dotted line: the value derived from the actual data (WT: N = 70; KO: N = 36).

(I) Histogram of p-values from Kolmogorov-Smirnov (KS) tests comparing WT and KO slopes in each phase-shuffled iteration (shuffling as in [H]). Red dotted line: the value derived from the actual data (WT: N = 70; KO: N = 36).

(J) As in (H), but for position shuffling, in which spike positions were randomly permuted within trials (WT: N = 70; KO: N = 36).

(K) As in (H), but for position shuffling as in [J] (WT: N = 70; KO: N = 36).

(L-M) Bootstrapping analysis to estimate the reliability of the observed group difference.

(L) Histogram of mean slope differences (WT minus KO) across 1,000 bootstrap iterations, resampling neurons with replacement while maintaining the original sample size per genotype (WT: N = 70; KO: N = 36).

(M) Histogram of p-values from Kolmogorov-Smirnov (KS) tests comparing WT and KO slopes in each bootstrap iteration (WT: N = 70; KO: N = 36).

(N) Cumulative probability distribution of the median field size for neurons included in the phase precession analysis. Gray shaded region: ± SEM for WT, centered at the mean; green shaded region: ± SEM for KO, centered at the mean (WT: N = 70; KO: N = 36; Kolmogorov–Smirnov test).

(O) Relationship between phase precession slope and log-transformed field size across neurons (WT: gray; KO: green; WT: N = 70; KO: N = 36).

bioRxiv preprint doi: https://doi.org/10.64898/2026.03.22.713468; this version posted March 22, 2026. The copyright holder for this preprint (which was not certified by peer review) is the author/funder, who has granted bioRxiv a license to display the preprint in perpetuity. It is made available under aCC-BY 4.0 International license.

(P) Relationship between phase precession slope and theta modulation strength (MVL; WT: gray; KO: green; WT: $N = 70$; KO: $N = 36$).
*$p < 0.05$; **$p < 0.01$, ****$p < 0.0001$.

bioRxiv preprint doi: https://doi.org/10.64898/2026.03.22.713468; this version posted March 22, 2026. The copyright holder for this preprint (which was not certified by peer review) is the author/funder, who has granted bioRxiv a license to display the preprint in perpetuity. It is made available under aCC-BY 4.0 International license.

Model	Predictors	Adjusted R^2	Coefficients	p-Values
1	Genotype only	-0.0050	β (intercept) = 1.8775<br>β (genotype) = 0.0716	p(intercept) = 3.6432e-43<br>p(genotype) = 0.6851
2	Theta MVL only	0.0323	β (intercept) = 2.2373<br>β (theta MVL) = -1.7200	p(intercept) = 1.8350e-31<br>p(theta MVL) = 0.0110
3	Field size only	0.2710	β (intercept) = -0.8943<br>β (field size) = 0.9223	p(intercept) = 0.0134<br>p(field size) = 2.4337e-13
4	Genotype + Field Size + Theta MVL	0.2784	β (intercept) = -0.6398<br>β (genotype) = -0.2154<br>β (field size) = 0.9191<br>β (theta MVL) = -0.8995	p(intercept) = 0.1149<br>p(genotype) = 0.1643<br>p(field size) = 1.3433e-12<br>p(theta MVL) = 0.1333
5	Genotype * Field Size + Genotype * Theta MVL	0.2785	β (intercept) = -0.1919<br>β (genotype, WT) = -1.3817<br>β (field size) = 0.7841<br>β (theta MVL) = -1.1387<br>β (genotype, KO:field size) = 0.3894<br>β (genotype, KO:theta MVL) = 0.2785	p(intercept) = 0.7117<br>p(genotype) = 0.1036<br>p(field size) = 8.1668e-07<br>p(theta MVL) = 0.1081<br>p(genotype:field size) = 0.1613<br>p(genotype:theta MVL) = 0.7764

Table S1. Linear regression models examining predictors of phase precession slope. Models were fit using MATLAB's fitlm function. Each model included different combinations of predictors: genotype (WT vs. KO), field size (log-transformed), and theta modulation (log-transform of the mean vector length). The purpose of these models was to determine which variables best account for variability in phase precession slope and to assess the unique contribution of each predictor. Adjusted R^2 values were used to compare model fit while penalizing for model
